# Training for object recognition with increasing spatial frequency: A comparison of deep learning with human vision

**DOI:** 10.1167/jov.21.10.14

**Published:** 2021-09-17

**Authors:** Lev Kiar Avberšek, Astrid Zeman, Hans Op de Beeck

**Affiliations:** 1 Department of Brain and Cognition, Leuven Brain Institute, Faculty of Psychology & Educational Sciences, KU Leuven, Leuven, Belgium; 2 Department of Psychology, Faculty of Arts, University of Ljubljana, Ljubljana, Slovenia; 3 Department of Brain and Cognition, Leuven Brain Institute, Faculty of Psychology & Educational Sciences, KU Leuven, Leuven, Belgium; 4 Department of Brain and Cognition, Leuven Brain Institute, Faculty of Psychology & Educational Sciences, KU Leuven, Leuven, Belgium

**Keywords:** human vision, spatial frequency analysis, deep convolutional neural networks, coarse-to-fine progression, computational modeling

## Abstract

The ontogenetic development of human vision and the real-time neural processing of visual input exhibit a striking similarity—a sensitivity toward spatial frequencies that progresses in a coarse-to-fine manner. During early human development, sensitivity for higher spatial frequencies increases with age. In adulthood, when humans receive new visual input, low spatial frequencies are typically processed first before subsequent processing of higher spatial frequencies. We investigated to what extent this coarse-to-fine progression might impact visual representations in artificial vision and compared this to adult human representations. We simulated the coarse-to-fine progression of image processing in deep convolutional neural networks (CNNs) by gradually increasing spatial frequency information during training. We compared CNN performance after standard and coarse-to-fine training with a wide range of datasets from behavioral and neuroimaging experiments. In contrast to humans, CNNs that are trained using the standard protocol are very insensitive to low spatial frequency information, showing very poor performance in being able to classify such object images. By training CNNs using our coarse-to-fine method, we improved the classification accuracy of CNNs from 0% to 32% on low-pass-filtered images taken from the ImageNet dataset. The coarse-to-fine training also made the CNNs more sensitive to low spatial frequencies in hybrid images with conflicting information in different frequency bands. When comparing differently trained networks on images containing full spatial frequency information, we saw no representational differences. Overall, this integration of computational, neural, and behavioral findings shows the relevance of the exposure to and processing of inputs with variation in spatial frequency content for some aspects of high-level object representations.

## Introduction

The role of spatial frequency has been extensively researched in the development of human vision, with much effort being directed toward visual acuity and contrast sensitivity (Banks & Salapatek, 1978; Benedek, Benedek, Kéri, & Janáky, 2003; Ellemberg, Lewis, Liu, & Maurer, 1999; Leat, Yadav, & Irving, 2009; Mayer & Dobson, 1982; Norcia & Tyler, 1985; Norcia, Tyler, & Hamer, 1990; Peterzell, Werner, & Kaplan, 1995; Stiers, Vanderkelen, & Vandenbussche, 2003). Visual acuity can be classified into recognition (perceived detail) and resolution (the separation between dots or gratings, or spatial frequency) that a person can successfully resolve. Contrast sensitivity is the smallest difference in luminance that can be perceived between an object and its immediate surroundings. Contrast sensitivity is measurable across the whole spectrum of spatial frequencies, referred to as the contrast sensitivity function (Leat et al., 2009). Researchers have used a variety of methods, including visual evoked potentials (Norcia & Tyler, 1985; Norcia et al., 1990) and psychophysiological methods (Banks & Salapatek, 1978; Benedek et al., 2003; Ellemberg et al., 1999; Mayer & Dobson, 1982; Peterzell et al., 1995; Stiers et al., 2003), to reach the conclusion that visual acuity and contrast sensitivity increase with age until adolescence. At an infant level, visual acuity and contrast sensitivity are very poor, and over the span of a number of years they improve to reach a high functioning level, usually 7 to 12 years for visual acuity and 8 to 19 years for contrast sensitivity (according to Leat et al., 2009). The peak of an infant's contrast sensitivity and the point at which the infant's contrast perception falls to zero (their cut-off frequency) are both lower than those of adults (Kiorpes, 2016; Leat et al., 2009).

In addition to this coarse-to-fine progression during the ontogenetic development of human vision, there appears to be a similar tendency in the real-time neural processing of fully mature adults as their visual systems receive input moment to moment. Modern theories of vision suggest that the visual system works in a coarse-to-fine manner, in which the low spatial frequency (LSF) content of visual input, which contains coarse information such as global shape, takes precedence over high spatial frequency (HSF) content, which is important for seeing the finer details (Kauffman, Ramanoël, & Peyrin, 2014). A number of neuroimaging studies in human cortical areas of scene and object processing provide support for this coarse-to-fine processing. According to Bullier (2001), faster LSF processing in the dorsal stream guides the slower HSF processing in the inferotemporal cortex. Categorization of visual stimuli may be dominated by LSF or HSF information, depending on the duration of the stimuli being presented (Schyns & Oliva, 1994). Activation of orbitofrontal cortex is elicited by stimuli containing LSF information, 50 ms prior to areas in the temporal cortex, but not by HSF-only images, indicating that orbitofrontal cortex has an important role in top–down facilitation of image recognition (Bar et al., 2006). LSF modulates HSF processing in broadband images, as measured by electroencephalography in human participants (Petras et al., 2019). When LSF is informative of the image content, HSF contributes to a lesser extent, indicating that coarse information does indeed guide the processing of fine detail.

Developmental progression from lower to higher spatial frequencies appears to be an inherent characteristic of the human visual system. Here, we explore whether this progression would alter the representations that emerge in image processing. Interestingly, in the past few years, a new class of models for human visual information processing has become very popular, namely deep convolutional neural networks (CNNs). With the unprecedented success of CNNs on tasks such as image recognition (for an overview, see He, Zhang, Ren, & Sun, 2015; Krizhevsky, Sutskever, & Hinton, 2012; LeCun, Bengio, & Hinton, 2015; Simonyan & Zisserman, 2015), these models are now widely used for tasks depending on visual representations in cognitive and computational neuroscience domains. Jozwik, Kriegeskorte, Storrs, and Mur (2017) found that CNNs outperform feature-based models in explaining human similarity judgments. Peterson, Abbott, and Griffiths (2017) demonstrated a high correlation between the activations in fully connected layers of CNNs and human behavioral similarity judgments of animal image pairs. Kubilius, Bracci, and Op de Beeck (2016) discovered a striking similarity between human behavioral and CNN shape representation. Bracci, Ritchie, Kalfas, and Op de Beeck (2019) found that CNNs classify objects by animacy rather than appearance, and this bias toward animacy is also demonstrated in human judgments and brain representations. Zeman, Ritchie, Bracci, and Op de Beeck (2020) found that CNNs represent category independently from shape in object images, similarly to human object recognition areas. Altogether, these studies and more have demonstrated the strength and breadth of applications for CNNs as a model of human vision.

Despite these promising results, some studies have shown intriguing counterintuitive properties of CNNs that place doubt on their viability as a model of the human visual system. For example, Jozwik et al. (2017) found that categorical models outperform CNNs in human similarity judgments, concluding that further improvements are needed to make high-level semantic representations more human like. Peterson et al. (2017) discovered that major categorical divisions (between animal images) were missing in CNN representations; multidimensional scaling showed that major categorical divisions were not preserved. Szegedy, Zaremba, Sutskever, Bruna, Erhan, Goodfellow, and Fergus (2013) observed that minute, imperceptible (for humans) changes to an image can drastically change the predictions by a CNN for that image, which surprisingly, and alarmingly, can generalize to models with different architectures that are trained under different procedures. Similarly, Nguyen, Yosinski, and Clune (2015) showed that CNNs can be easily fooled by images generated using an evolutionary (gradient ascent) algorithm. These images, which are unrecognizable to human observers, are categorized by CNNs with a very high confidence level and are referred to as adversarial examples. Although there is some evidence that human classification of adversarial examples under forced-choice condition is robustly related to machine classification (Zhou & Firestone, 2019), there is little explanation of why such convergence occurs. Also, Dujmović, Malhotra, and Bowers (2020) found that agreement between humans and CNNs on adversarial examples is much weaker and more variable than that reported by Zhou and Firestone (2019). According to Wang, Wu, Huang, and Xing (2020), the vulnerability of CNNs to adversarial examples might be a consequence of their over-reliance on high spatial frequency information. Several studies (e.g., Baker, Lu, Erlikhman, & Kellman, 2018; Geirhos, Rubisch, Michaelis, Wichmann, & Brendel, 2019) have also shown a texture bias in CNNs, indicating that texture, which might be carried mostly by HSF information, is predominantly used by artificial networks to classify objects, which is opposite to the human classification strategy, in which shape is the primary cue. Given these findings, it is clear that there are further improvements to be made with CNNs, not only in their architectural components and connectivity but also in the method in which they are trained. One of the relevant dimensions is the spatial frequency content of images. For example, some of the aforementioned discrepancies between human and deep neural network representations might be due to a differential sensitivity for spatial frequency. This hypothesis was mentioned by Wang et al (2020) but has not been tested explicitly for most of these discrepancies.

In spite of the convolutional and pooling layers of CNNs being directly inspired by simple and complex cells in visual neuroscience and that the overall architecture is reminiscent of the lateral geniculate nucleus–visual cortex 1 (V1)–V2–V4–inferior temporal cortex hierarchy in the visual ventral pathway (LeCun et al., 2015), the idea of progressively increasing the spatial frequency content of images during training has not yet been implemented. One notable exception was implemented for generative adversarial networks, initially training with low-resolution images before continuing with higher resolution images (Karras, Aila, Laine, & Lecthinen, 2018). This progressive method allowed for these networks to “discover the large-scale structure of images prior to details at increasingly finer scales, as opposed to learning all scales simultaneously” (Karras et al., 2018, p. 2). The training benefits were twofold with regard to sufficiently decreasing the training time and improving the stability in synthesizing both low- and high-resolution images. Although such a training regime demonstrated clear benefits, we note that this study did not include any conjecture or comparison with human vision, so the question remains as to whether training CNNs under such conditions would bring about better performance changes or representational changes that would increase their similarity to human observers.

Following on from the culmination of findings from different fields, we hypothesized that training a CNN by simulating the progressive exposure from low to high spatial frequencies would increase the performance to be similar to that shown by human behavior, in addition to increasing the similarity between artificial and human visual representations. By encouraging the environmental conditions in which an artificial visual brain would develop to be more similar to that of a biological visual brain, we may potentially overcome some of the current limitations of CNNs as models for human visual functions. In the present study, we extensively analyzed the effects of implementing a training protocol for deep neural networks that progressively increases the spatial frequency information of images. We compared our results against a variety of human findings, including behavioral and neuroimaging studies.

## Method

### CNN implementation

For our model architecture, we selected MobileNet (Howard et al., 2017), an efficient, serially connected CNN with 28 layers. We trained MobileNet on a subset of ImageNet, used in the ImageNet Large Scale Visual Recognition Challenge (ILSVRC) 2012–2017 image classification and localization dataset (Russakovsky et al., 2015), which contains approximately 1.2 million images from 1000 classes. We implemented three different training regimes (see Table 1):

1. *Full training*—We trained the network using unfiltered images, in which no spatial frequency (SF) information was omitted from the images. This regime is the most commonly used technique and served as a control condition.
2. *Gradual training*—We progressively shifted the SF information of images during training. At every 100th epoch, the training set was switched to a set of identical, albeit differently filtered, images. There were five different training sets, which included one unfiltered set and four differently filtered training sets, each containing images with higher SF information than the previous (see “Filtering of images” section).
3. *Mixed training*—Taking an approach similar to the gradual-training method, we changed the training set every 100 epochs, except that a proportion of images from the previous set were retained in the subsequent set (those containing lower SF information).

**Table 1.** Training regimes of MobileNet. Values below the decimal number (c/°) and UF columns denote the proportion (%) of images at the given stage of training. *Note:* LR = learning rate; UF = unfiltered.

	Full training						Gradual training						Mixed training					
Epoch	LR	0.1	0.3	0.5	0.8	UF	LR	0.1	0.3	0.5	0.8	UF	LR	0.1	0.3	0.5	0.8	UF
100	0.5	0	0	0	0	100	0.5	100	0	0	0	0	0.5	100	0	0	0	0
200	0.5	0	0	0	0	100	0.5	0	100	0	0	0	0.5	50	50	0	0	0
300	0.5	0	0	0	0	100	0.5	0	0	100	0	0	0.5	25	25	50	0	0
400	0.1	0	0	0	0	100	0.1	0	0	0	100	0	0.1	16	16	16	50	0
450	0.01	0	0	0	0	100	0.01	0	0	0	0	100	0.01	12.5	12.5	12.5	12.5	50

MobileNet converged in 500 epochs (batch size = 192, steps per epoch = 100). We used stochastic gradient descent with Nesterov momentum (0.9) as an optimizer and categorical cross-entropy as a loss function. The initial learning rate was 0.5, which decreased to 0.1 after 400 epochs and decreased again to 0.01 after 450 epochs. We measured validation accuracy using validation images that were also filtered on the same SF levels as the training sets. All training regimes were implemented in TensorFlow.

### Filtering of images

We filtered the train and test images in MATLAB (MathWorks, Natick, MA) using low-pass Butterworth filters (Butterworth, 1930) by executing the following procedure. First, images were resized to 224 × 224 × 3, which is the training size for MobileNet. Second, to convert the measuring unit of spatial frequency information (cycles per degree) into an analogous unit used by the Butterworth filters (radius in the frequency domain), we defined the pixels per cycle for given images. We assumed a visual angle of 60°, which is the visual angle of a standard smartphone (Apple iPhone 6). Knowing the dimensions of images (224 × 224 × 3) and having a physical measure (60°), we could approximate pixels per degree (224/60). This allowed us to compute SFs in cycles per degree (see Equation 1). Radius in the frequency domain was then compared to cycles per degree, providing us with all the required prerequisite information to apply Butterworth filters. Third, we applied four different Butterworth filters to ImageNet, each one with attenuating spatial frequencies higher than a threshold in cycles per degree. The thresholds that we applied, referred to as spatial frequency levels (SFLs), were 0.1 c/° (SFL1), 0.3 c/° (SFL2), 0.5 c/° (SFL3), and 0.8 c/° (SFL4). The specific values of filters were chosen to mimic the development of infant contrast sensitivity levels found in different studies (see Kiorpes, 2016). The four thresholds represent the approximate peak levels of the contrast sensitivity function at different stages of development, from infancy to 8 months, which is considered to be the most intense period in development. Equation 2 defines the Butterworth filter, where *D* is distance from the center (namely, 113 pixels, given an image size of 224 × 224), *r* is the radius in the frequency domain, and *n* is the order of the filter (all filters were of order 4):

(1) $$\text{SF}=\left(\frac{\;\text{pixels}\;/\;\text{degree}\;}{\text{pixels}\;/\;\text{cycle}}\right)$$

(2) $$\text{Filter}=\frac{1}{1+\left(\sqrt{2}-1\right)\times {\left(\frac{D}{r}\right)}^{\left(2\times n\right)}}$$

### Representational similarity analysis (RSA)

After training the CNNs, we compared representations between them using RSA, which is a framework that allows for quantitative comparisons of internal representations between computational models and even other modalities, such as neural activity and behavior (Kriegeskorte, Mur, & Bandettini, 2008). For CNN representations, after forward passing the images through the network, we extracted activations from the final fully connected layer and a mid-level convolutional layer (layer 15), in each of the three implementations of MobileNet. From these activations, we constructed representational dissimilarity matrices (RDMs). RDMs represent dissimilarities of activations for each image by measuring correlations (1 – Spearman's rho). We then compared the RDMs (using Spearman's rho) to the behavioral and conceptual RDMs from the mentioned studies.

To test for the effect of spatial frequency on representational similarity, we also constructed a set of hybrid images, which were inspired by Schyns and Oliva (1994). These images consisted of two different superimposed images, one filtered with a low-pass filter (<0.17 c/°) and one with a high-pass filter (>0.17 c/°). The set was composed of 18 images. Nine of those images, labeled HSF∆, consisted of the same LSF content but different HSF content. The other nine images, labeled LSF∆, consisted of the same HSF content but different LSF content. We composed two conceptual RDMs for the set of hybrid images, the so-called LSF and HSF models. We postulated that, if an implementation of MobileNet is sensitive only to the HSF content of images, it should represent the images where LSF content is manipulated as more similar than those where HSF content is manipulated (HSF model). In contrast, if an implementation of MobileNet is sensitive only to the LSF content of images, it should represent the images where HSF content is manipulated as more similar than those where LSF content is manipulated (LSF model).

To detect any possible changes in representations due to our training protocol, we included two stimulus sets along with the associated CNN, behavioral, and neural similarity data from Bracci et al. (2019) and Bracci and Op de Beeck (2016), with further analyses by Zeman et al. (2020), for comparison with our trained networks. In both studies, the authors dissociated appearance from the category for each of the stimuli, allowing for a controlled comparison between visual and more conceptual information (see Figures 2 and 3).

We computed inferential statistics using random permutations and bootstrap methods. To examine if a model RDM correlated significantly with a target (conceptual or behavioral) RDM, we permuted image labels 10^4^ times. We then correlated all the permuted RDMs with the target RDMs. We calculated the *p* value by taking the number of correlations with a greater value than the correlation of the model and dividing this by the number of all possible correlations (10^4^). The error bars shown later in Figure 7 depict the standard deviations from bootstrapping the correlation of each model 10^4^ times.

### Behavioral experiment

To assess human performance in image classification using differently filtered images, we constructed a behavioral experiment in psychopy (Peirce et al., 2019). We chose 10 categories with consistent performance in the trained CNNs by extracting activations from the classification layer after feeding in the images from each category and confirming that accuracy levels were within 15% of average performance (15% above or below). These were hammerhead shark (H), cock (C), badger (B), dial telephone (D), planetarium (P), sports car (S), upright piano (U), acorn squash (A), Granny Smith apple (G), and red wine (R). The experiment consisted of two stages. In the first stage, the training phase, participants viewed 10 unfiltered examples of each category in a randomized order. An image was displayed indefinitely, and participants were required to press the letter of the corresponding category in order to continue.

The testing phase followed. In this phase, participants were presented with images in a randomized order from the 10 categories with three spatial frequency cutoffs: 0.1 c/° (SFL 1), 0.3 c/° (SFL 2), and no cutoff (unfiltered). Each image was presented for 150 ms. After the elapsed time period, the participant was required to select the letter corresponding to the image (e.g., “A” for acorn squash). Participants classified 30 images per category, with 10 per spatial frequency level (for a total of 300 images). All images and exemplars were shown only once. To compare human performance in the 10-way categorization task with CNN performance, we extracted the activations from the classification layer only for the 10 categories. Categorization was correct if the activation of the corresponding category had the highest value over the other nine possible outcomes (referred to as “top-1 accuracy,” in this case for a choice among 10 categories instead of the 1000 categories in ImageNet). Note that we could not control each participant's viewing distance, which can be seen as a caveat, as this can affect the appearance of the images. However, this experiment was concerned with how much information was left on the images, rather than about mimicking the exact spatial frequency and stimulus size we targeted when we decided on the filtering levels.

The experiment included 28 participants (11 males, 15 females, and 2 “other”), with an average age of 23.54 years (*SD* = 3.13), who participated through the online platform Pavlovia.com. The experiment included an agreement to ensure informed consent prior to testing. Procedures were approved by the KU Leuven Social and Societal Ethics Committee.

## Results

### Training and validation accuracy with the three training regimes

The MobileNet trained with the full training converged to the highest training accuracy (74%) and validation accuracy (60%) for unfiltered images (Figure 6). However, it performed very poorly (0%) on the lowest spatial frequency level SFL 1 (0.1 c/°). Accuracy increased with images containing higher spatial frequency information (21% for SFL 2, 36% for SFL 3, and 48% for SFL 4). With gradual training, MobileNet showed increasingly better performance during learning for the first 100 epochs, during which it was exposed to only low SF images (SFL 1). However, there was an immediate drop (to 0%) in performance after switching to the image set containing higher SF information. Simultaneously, there was a very rapid stepwise increase of accuracy, from 0% to around 50%, for the sets containing higher SF information. With mixed training, we again observed a very rapid increase in performance of MobileNet after the 100th epoch for images containing higher SF information; yet, there was no drop in performance for images containing the lowest spatial frequency information, which instead converged further. Interestingly, the mixed training policy was the only training regime that allowed the model to reach a sufficient level of performance on the images containing only the lowest SF information (32%) while maintaining relatively high levels of accuracy on the images with higher cutoff levels and unfiltered images (48% for SFL 2, 50% for SFL 3, 51% for SFL 4, 60% for training accuracy, and 51% for validation accuracy on unfiltered images).

### Comparison with human performance for low-pass filtered images

We tested how well human participants would classify low-pass filtered images; the results are shown in Figure 7. Note that participants did not receive training with such images in the context of the experiment. As the behavioral experiment included only 10 categories, the performance of the CNNs was also calculated for the same 10-category task. If we consider the most strongly filtered images as SFL 1, the full-training policy, which was never presented with these filtered images during training, performed very poorly. Although the gradual training was initially trained on filtered images, its performance on SFL 1 did not exceed that of the full-training policy. Both human participants and the mixed training model reached relatively high accuracy with the SFL 1 images, the latter surpassing human performance by about 10%. With the SFL 2 condition, the full training showed a marked drop in performance compared with unfiltered images that was not present in the other models and in human performance.

Overall, it seems that an extensive and continuous exposure to filtered images during training is necessary to allow deep learning models to reach the capacity of humans to recognize images that contain only low spatial frequency content.

### RSA with hybrid stimuli

We investigated whether the training affected the representational similarity of the hybrid images that combine different information at low and high spatial frequencies. The results are shown in Figure 8. Both layers show similar effects of the type of training. This suggests that the differentiability that the networks learn to distinguish images with low SF content is present in earlier layers of the network and not only in the final classification layer.

Figures 9 and 10 display the correlations of these CNN representations with the conceptual LSF and HSF model. In the fully connected layer, only the model trained with the mixed-training regime reached significant positive correlations for both the LSF and HSF models. In other words, the mixed training model was sensitive to the manipulation of both the low and high spatial frequency content of the images. In contrast, the full-training model had a significantly negative correlation with the LSF model and the highest positive correlation with the HSF model. This indicates that, after full training, the network is capable of differentiating images with varied HSF content but not images with varied LSF content, which elicit similar activations. Findings were similar between the convolutional layer and the fully connected layer, with the exception that in gradual training the convolutional layer correlated with the LSF model. Overall, a mixed-training regime is required for a model to become sensitive to both the low and high spatial frequency information in images.

### RSA with stimuli from Bracci and Op de Beeck (2016) and Bracci et al. (2019)

To this point we have shown that a mixed-training regime is important to obtain a CNN that is able to recognize objects from LSF content and take this content into account in object representations. Next, we investigated whether such a training regime would also affect the representational similarity for stimulus sets that do not include an explicit manipulation of spatial frequencies.

For the stimulus set of Bracci and Op de Beeck (2016), all three training regimes of MobileNet preferred shape over category. In addition, they also showed a smaller, yet significant correlation with the category-related behavioral similarities, as was shown by Zeman et al. (2020) for other networks trained with unfiltered images. There were no meaningful effects of the training regime, so the extent and timing of training with low-pass filtered images did not affect the presence of a multi-feature representation in a CNN.

Bracci et al. (2019) showed that CNNs pretrained on ImageNet (VGG-19 and GoogleNet) had a strong bias for the animacy model over the appearance model, which puts CNN models at odds with human perception and neural responses. We also see a stronger correlation for the animacy model with MobileNet when trained with unfiltered images. Nevertheless, the bias toward animacy in the late fully connected layers was much larger in Bracci et al. (2019).

The bias toward animacy was no longer present when training included low-pass filtered images. Given the variability (shown by large error bars), we cannot state that this effect is significant, so the findings are inconclusive with regard to a meaningful effect of training regime on the nature of representations investigated with this stimulus set.

## Discussion

In our study, we thoroughly examined the effect of a training regime with progressive exposure to images with increasingly finer spatial frequencies, akin to biological vision. This training method allowed us to determine the effect on deep neural network performance and representations and to investigate whether this effect resembled human performance and representations.

The most widely used training regime in computer vision and neuroscience communities, which exposes a model only to unfiltered images, is not able to accurately classify visual stimuli that contain only low spatial frequency information. In both the 1000-way and the 10-way task (Figures 6 and 7), the full-training model demonstrated very low levels of categorization accuracy when presented with images that had a cutoff spatial frequency of 0.1 c/° (SFL 1). Comparing this outcome with human performance, participants demonstrated much greater accuracy with such stimuli, surpassing the performance of the full-training model by almost a factor of 3, despite most of the spatial frequency information being omitted (SFL 1). Notably, even with initial exposure to SFL 1 images, the performance of the model on these images was not sustained unless a proportion of those images were retained in the training set. This was visible in the performance of the gradual-training model (Figure 6), with an immediate drop after switching to images with a higher cutoff (SFL 2). We observed a similar, albeit less dramatic, fall in performance for SFL 2 images at epoch 200. There was no apparent drop in accuracy for higher cutoff levels (SFL 3 and 4). On the other hand, the mixed-training model was able to sustain relatively high levels of accuracy across the entire spatial frequency spectrum. Furthermore, it produced results in the 10-way categorization task that highly resemble the performance of human participants.

Our findings with gradual training are peculiar. Given the cumulative nature of spatial frequency information, where each set of images with a higher SF cutoff contains information from the lower SF cutoff plus new, higher SF information, we would expect that initial exposure is enough to calibrate the weights of a model in a way that would later provide sensitivity to low spatial frequencies. However, this was not the case. Apparently, providing images with lower levels of spatial frequency information is necessary throughout the entire training process to sustain high levels of accuracy. This could be a result of the non-additive way in which weights are computed in the models. From this perspective, the relationship between the nonlinear functions of the model with those of the human visual system is put into question. Although CNNs reach high levels of accuracy in image categorization tasks, they might achieve this in an inherently different way as humans. To express it another way, the ability to achieve similar results, in terms of outstanding categorization accuracy, might stem from different underlying processes.

Hermann and Lampinen (2020) examined some of the underlying processes of feature extraction in CNNs. They found that when trained to label based on a target feature (either global information as shape or local information as texture), CNNs were able to enhance the target and suppress the irrelevant feature. Furthermore, when two features were redundantly predictive of the label, the model extracted one and suppressed the other. In their experiment, shape had precedence over texture. Thus, the feature extraction process seemed to engage in a sort of “zero-sum game,” the outcome of which depended on the final goal that was set for the model and the resulting redundancy of specific features. Drawing a parallel to the gradual-training protocol, we could assume that the sudden decrement in classification accuracy for images containing lower SF information could be a result of (global or LSF) information suppression, because this information became redundant after the goal of the model changed to classifying images with higher SF information. Such processes might not occur in the human visual system; rather, the human visual system utilizes LSF information early in the process to guide the recognition of an object (Bar et al., 2006; Bullier, 2001; Kauffman et al., 2014; Petras, Oever, Jacobs, & Goffaux, 2019; Schyns & Oliva, 1994).

**Figure 1.**
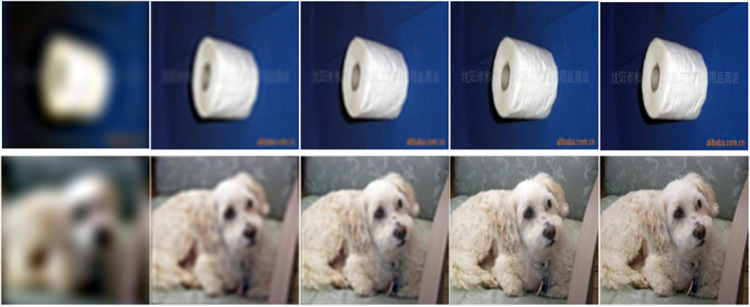
Example of two images from ImageNet with the Butterworth filter applied using increasing SF cutoff thresholds. From left to right, 0.1 c/° (SFL 1), 0.3 c/° (SFL 2), 0.5 c/° (SFL 3), 0.8 c/° (SFL 4), and the original unfiltered image.

The possibility of divergence between the underlying processes of the human visual system and of CNNs is further supported by the findings with the full training. Even for the SFL 2 images, the accuracy of the full-training model was substantially lower than that of the human participants, whose accuracy was almost equivalent to the unfiltered images (Figure 7). Indeed, if we look at an example of an SFL 2 image (Figure 1^2^,^3^,^4^,^5^,^6^,^7^,^8^), the changes caused by the filter are so subtle that they are barely registered by the human eye. Yet, the full-training model struggled with such images. These limitations were solved using a simple technique of retaining the lower SF images in the training set the entire time, as we did in the mixed-training regime. These findings are consistent with the observations of Wang et al. (2020). In their paper, they suggested that counterintuitive generalizations of CNNs (such as adversarial examples) might be the result of excessive reliance of the models on HSF information and can be dealt with by annulling these signals with smooth convolutional kernels. The hypothesis that CNNs rely primarily on HSF information is also supported by Baker et al. (2018). They found that, for CNNs, surface texture (local or HSF information) is an equally important cue in object recognition as shape (global or LSF information). Similarly, Geirhos et al. (2019) found texture bias in images with conflicting shape and texture. The equivalence, or even dominance, of the informative value of texture does not seem to hold for humans, who can accurately categorize objects by shape alone. These observations can be linked with our work if we assume that texture information is mostly carried by higher spatial frequencies. There is some evidence that supports this assumption. Namely, Yoshihara, Fukiage, and Nishida (2021) were able to induce a shape bias in CNNs by training them with blurred (LSF) images, although the bias was still much smaller than in human observers.

**Figure 2.**
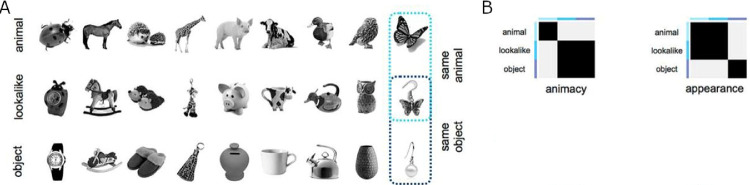
Stimuli (A) and conceptual models (B) based on Bracci et al. (2019). The black areas in the RDMs represent high similarity (1 – Spearman's rho = 1), and the gray areas represent low similarity (1 – Spearman's rho = 0). To construct the RDMs, stimuli were numbered left to right, top to bottom.

**Figure 3.**
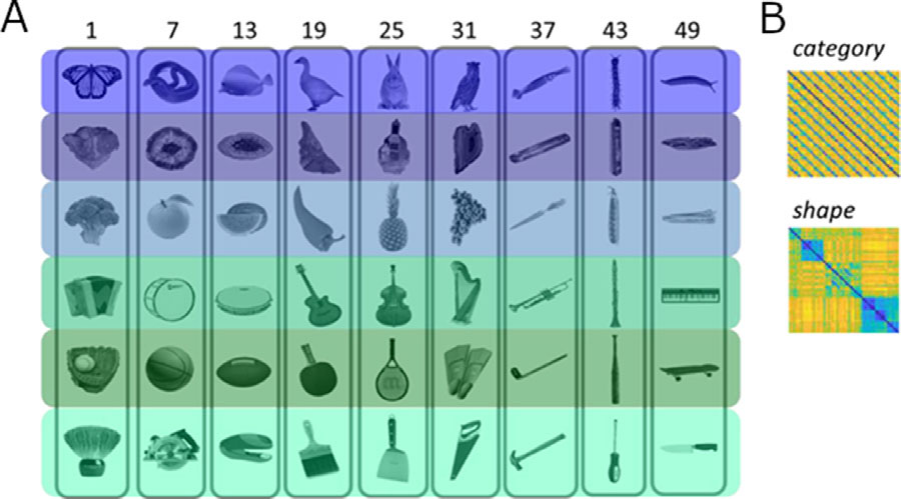
Stimuli (A) and behavioral models (B) based on Zeman et al. (2020). The rows in (A) represent stimuli that share category but differ in shape, whereas the columns represent stimuli that are similar in shape but belong to different categories. The colors represent the category organization. The behavioral matrices (B) use the color blue to represent high similarity, whereas yellow represents low similarity.

**Figure 4.**
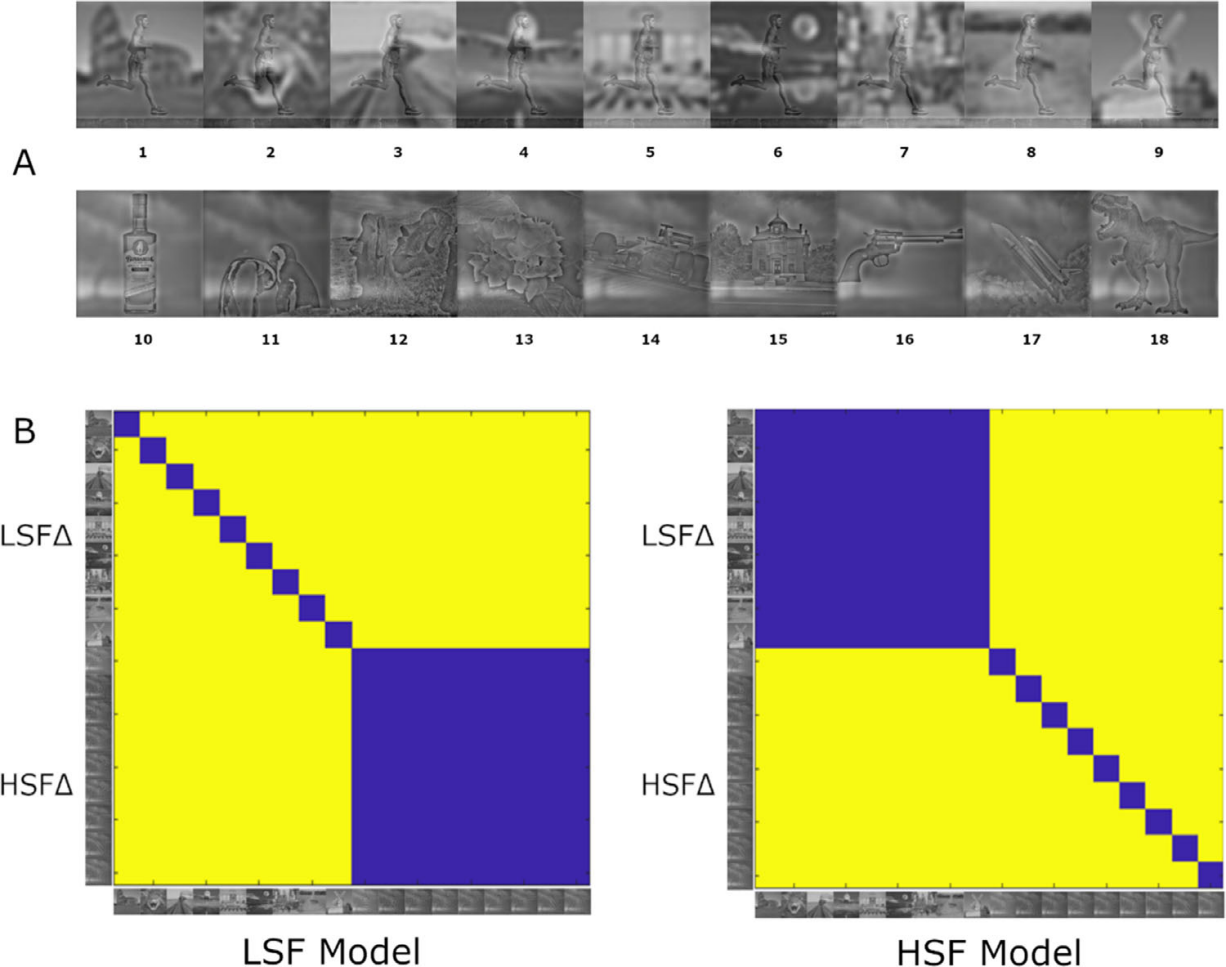
(A) Hybrid stimuli. The top row shows LSF∆ stimuli, and the bottom row displays HSF∆ stimuli. The numbering of images follows the top–bottom and left–right sequence in the RDMs. (B) Our conceptual RDMs. The matrix on the left in (B) represents the LSF model. The LSF model showed the greatest similarity between images that contained the same LSF content with varying HSF content and showed the lowest similarity between images with varying LSF but consistent HSF content. The matrix on the right in (B) represents the HSF model. The HSF model showed the greatest similarity between images that contained the same HSF content with varying LSF content and showed the lowest similarity between images with varying HSF but consistent LSF content.

**Figure 5.**
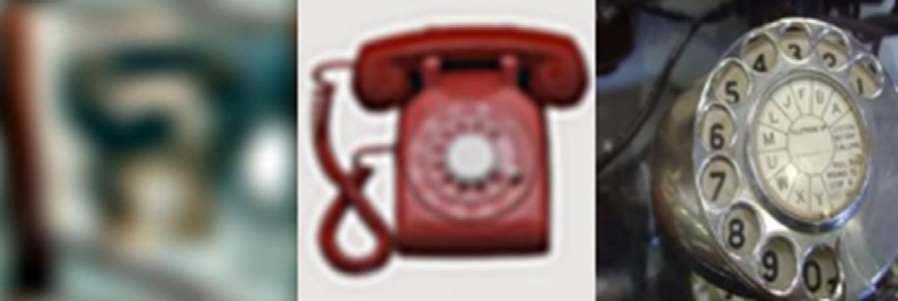
Example of SFL1, SFL2, and unfiltered images for the category “dial telephone.”

**Figure 6.**
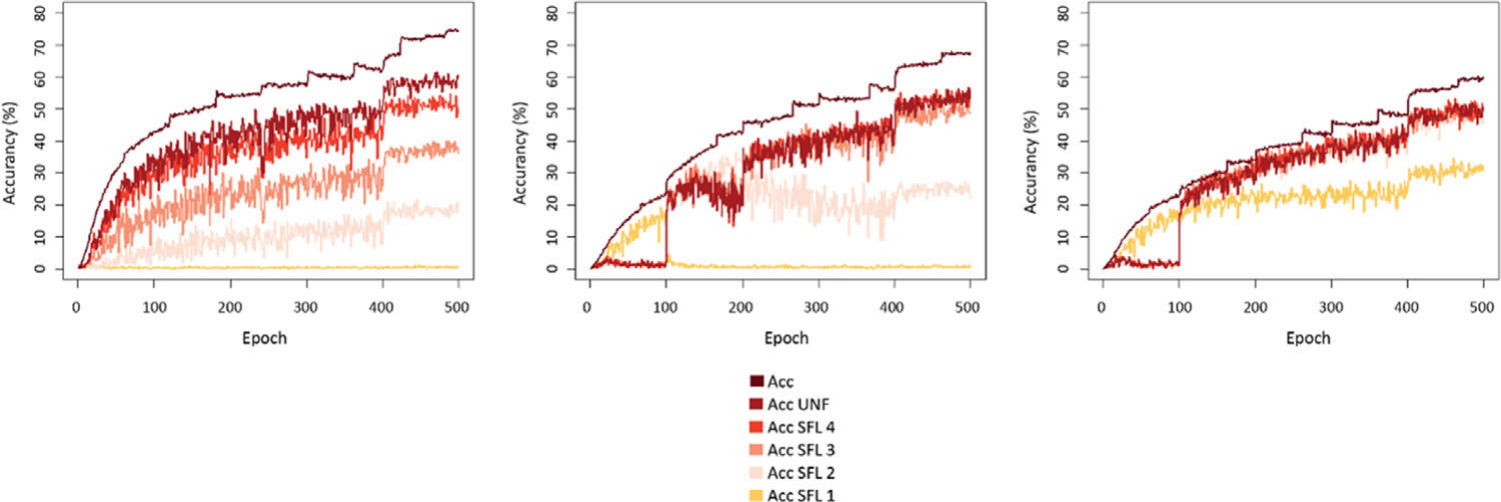
Top-1 performance of MobileNet trained with the full, gradual, and mixed (from left to right) regimes on images with different spatial frequency levels (SFL 1 = 0.1 c/°, SFL 2 = 0.3 c/°, SFL 3 = 0.5 c/°, SFL 4 = 0.8 c/°). Acc UNF = validation accuracy on unfiltered images, Acc = training accuracy.

**Figure 7.**
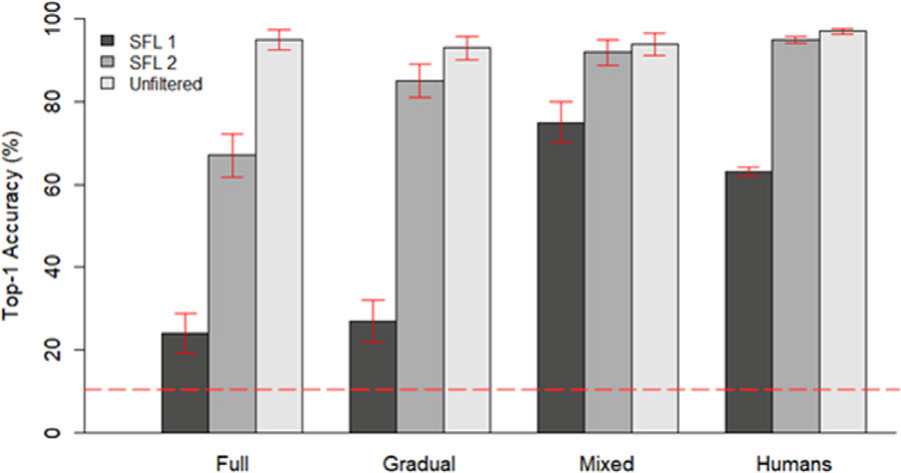
Performance of MobileNet trained with different regimes and humans on a 10-way categorization task containing images with different spatial frequency information (dark gray, SFL 1 = 0.1 c/°; mid-gray, SFL 2 = 0.3 c/°; light gray, unfiltered). In the case of MobileNet, error bars indicate the binomial confidence interval, whereas with humans they indicate standard error of the mean (*SEM*). The horizontal dashed line indicates the chance level (10%).

**Figure 8.**
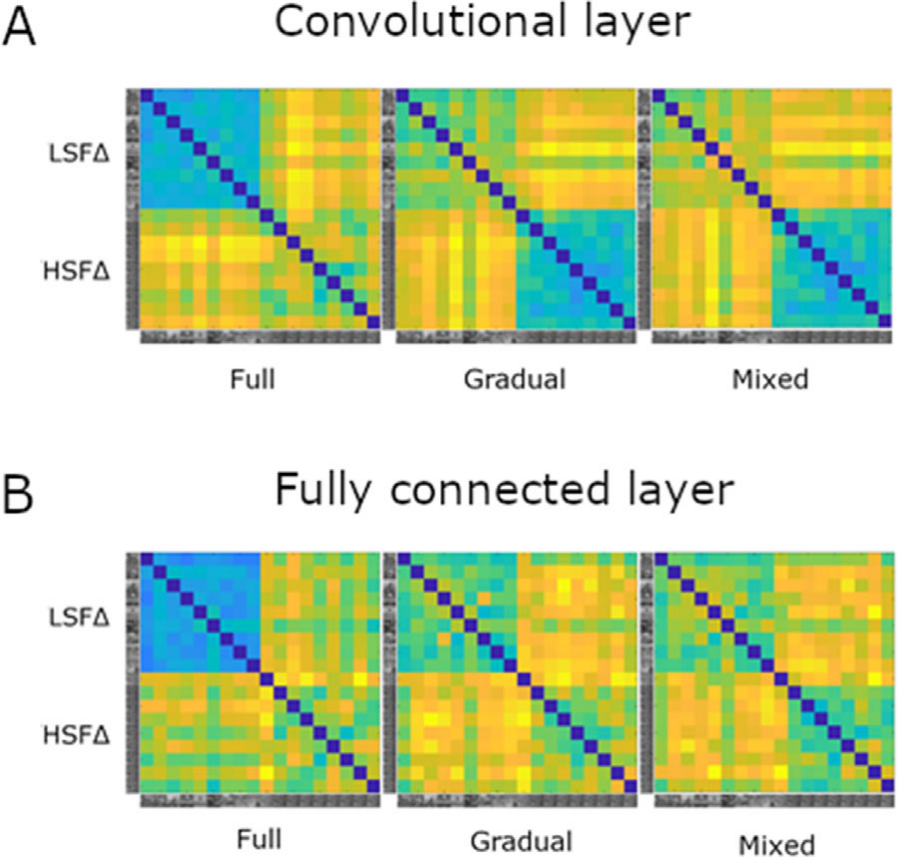
From left to right, RDMs for the full, gradual, and mixed training of MobileNet for the mid-level (layer 15) convolutional layer (A) and the fully connected layer (B). Blue denotes high similarity, whereas yellow denotes low similarity. Labels on the left side show how the stimuli were ordered.

**Figure 9.**
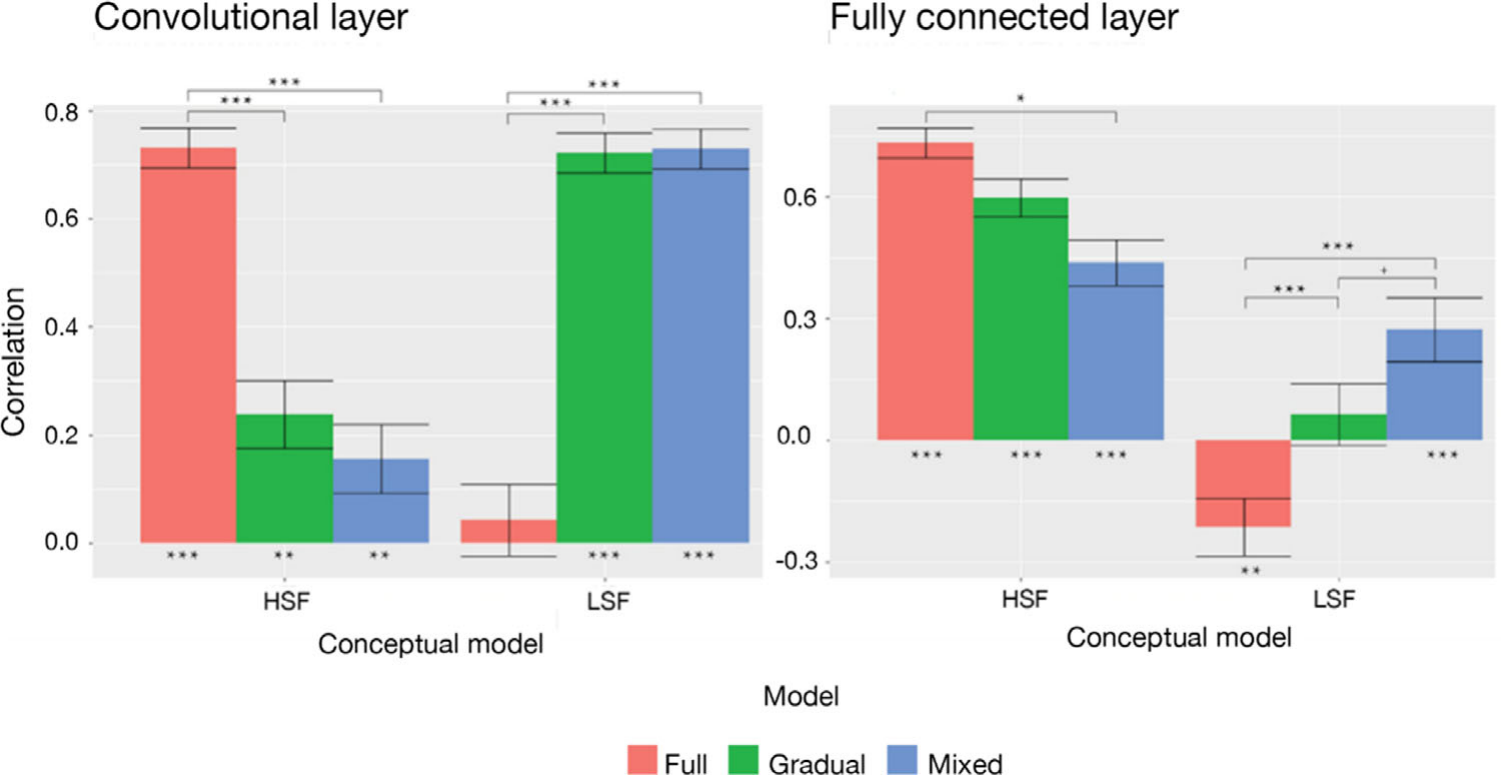
(Left) Correlations between conceptual models for hybrid stimuli and the convolutional layer (layer 15) of different MobileNet models (red, full training; green, gradual training; blue, mixed training). (Right) Correlations between conceptual models for hybrid stimuli and the fully connected layer of different MobileNet models. Standard errors represent the standard deviations of 10^4^ bootstraps. ^***^p < 0.001, ^**^p < 0.01, ^*^p < 0.05, ^+^*p* < 0.10.

The implication that the full-training model was not sensitive to LSF content was reaffirmed by results from RSA. We can see that the full-training model was sensitive to manipulation of HSF content but not LSF content, as it correlates significantly only with the HSF model. On the other hand, the mixed-training was sensitive to both manipulations, which is reflected in significant correlations with both the LSF and HSF models (Figure 9). We must acknowledge that there could possibly be a trade-off between sensitivities at different SF levels. The enhanced sensitivity for LSF information results in deteriorated sensitivity for HSF information. Thus, the full-training model surpasses the mixed-training model in terms of classification accuracy for unfiltered images in the 1000-way task and in correlation levels with the HSF model, where we can see an almost linear decrease in the full–gradual–mixed order (Figure 9). Nevertheless, this trade-off does not have such a large magnitude, and the mixed-training model produces very sensible behavior, incorporating both LSF and HSF information.

**Figure 10.**
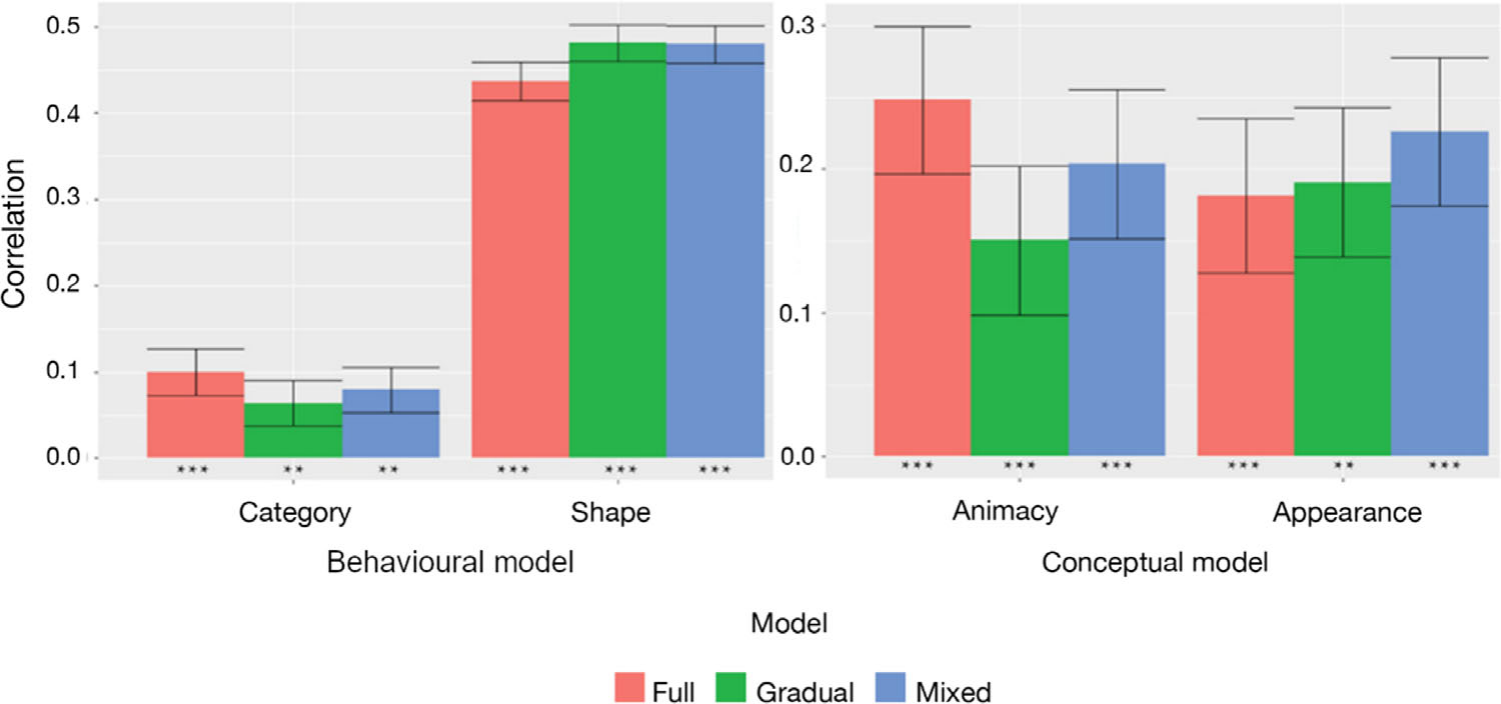
(Left) Behavioral model (Zeman et al., 2020) correlations with the fully connected layer of different MobileNet models. (Right) Conceptual model (Bracci et al., 2019) correlations with the fully connected layer of different MobileNet models. Standard error bars represent the standard deviations of 10^4^ bootstraps. ^***^p < 0.001, ^**^p < 0.01, ^*^p < 0.05, ^+^*p* < 0.10.

Furthermore, we examined if different training regimes affect internal CNN representations of visual stimuli, where spatial frequencies were not directly manipulated (Figure 10). Correlations with RDMs from Zeman et al. (2020) did not show any significant changes. All three models correlated significantly with both behavioral models but preferred shape over category. No statistically significant differences between models were found. Likewise, the results for Bracci et al. (2019) stimuli showed significant correlation for both conceptual models. There are some apparent fluctuations across training regimes, but given the standard errors we cannot conclude that there are significant differences. The findings with the full-training model tend to be most similar to the findings reported by Bracci et al. (2019), who demonstrated a much greater preference for the animacy model than for the appearance model. We could still see a slight preference for animacy with the full-training model, whereas this preference disappeared in the mixed-training model. Further tests with larger stimulus sets will be needed to investigate whether such effects would be statistically robust.

To sum up, we showed that CNNs trained in the usual way displayed some properties that make their similarity to human representations questionable. Firestone (2020) argued that, when comparing human to CNN behavior, we should make a distinction between competence and performance, this distinction being a methodological tool to compare the behavior of different organisms (e.g., infants and adults, animals and humans). Competence relates to the underlying capabilities of an organism, whereas performance relates to behavioral output, which does not necessarily reflect the full capacity of organisms’ underlying resources. Such an approach can facilitate differentiation between organisms at superficial and deep levels. Differences in behavior can be the result of a variety of factors. These include human constraints, machine constraints, and non-aligned species-specific tasks. One reason why the full-training regime performed so badly on LSF images might have been a limitation due to the digital input resolution. Humans view images on displays, using their lens, which can further distort the resolution (Firestone, 2020). In our case, the human constraint—limited visual acuity—could play a vital role in incorporating LSF information for object categorization. Mimicking human physiology in computational models has shown some promise before. For example, Adeli, Vitu, and Zelinsky (2017) showed that a model of superior colliculus can predict fixation locations in human viewing of natural scenes and exemplar and categorical search tasks. Even more interestingly, modeling a human fovea (Deza & Konkle, 2020) or primary visual cortex (Dapello, Marques, Schrimpf, Geiger, Cox, & DiCarlo, 2020) at the front of CNNs has been shown to increase their robustness to adversarial examples. Note that adversarial examples usually include subtle changes to images at high spatial frequencies. The human burden of limited visual acuity makes these manipulations unperceivable; thus, they do not affect human behavior (Firestone, 2020). It would be interesting to examine whether the addition of a foveal model or primary visual cortex would improve the capability of CNNs to use LSF information.

Similarly, we can ask whether our mixed-training regime would improve the resistance of CNNs to adversarial examples. As already mentioned, like humans, the mixed-training regime was capable of using LSF information and thus showed much higher (human-like) accuracy for such images than the full-training regime, which was trained by the method that is normally used in computational neuroscience. This technique was applied by keeping a proportion of images with a lower cutoff frequency filter throughout the entire training. Such a method restrained the model from only focusing on high spatial frequencies. In this way, we induced a “human-like” constraint, similarly to what Deza and Konkle (2020) did with adding a model of the human fovea. It also resembles the suggestion of Wang et al. (2020), who proposed annulment of HSF signals by smooth convolutional kernels. As Jang, McCormack, and Tong (2021) implied, the robustness to visual noise in CNNs is, like in humans, acquired through exposure and learning from noisy images.

Despite the similarity in performance, the question of concordance between the underlying processes remains. To elaborate on this point, Bar et al. (2006) demonstrated that LSF information affected the processing of HSF information in a top–down manner. None of our models was made to simulate top–down processes, which require recurrent connections. An intriguing pathway of research would be to implement our method of mixed training on a recurrent neural network. A further concern is the problem of generalizability. Our training protocols were implemented on a specific CNN (MobileNet) with a specific architecture and other properties. However, given that all CNN networks are very similar in how image content is treated in the convolutional layers, we expect that the major benefit of mixed training would generalize widely across networks, with possibly some variation in the effect size of this benefit.

Our findings provide some direct implications for real-world applications, particularly in the field of autonomous driving with incorporated real-time processing of camera images. According to Zang, Ding, Smith, Tyler, Rakotoarivelo, and Kaafar (2019), one of the critical issues of autonomous driving systems is their performance under adverse weather conditions, such as rain, snow, or fog. These conditions distort the pixel intensities and thus lower the quality of images. For example, raindrops can create patterns that blur the edges in a scene and, in doing so, impede the recognition of objects (Kurihata, Takahashi, Ide, Mekada, Murase, Tamatsu, & Miyahara, 2005). Of special interest is the perception of scenes under foggy conditions. Whereas scenes are composed of a broad spectrum of spatial frequencies under normal viewing conditions, the frequency components are concentrated at low spatial frequencies under foggy conditions (Zang et al., 2019). Thus, fog acts like a low-pass filter that blurs the finer details of an image. As we have shown, such a filter can have detrimental effects for deep neural networks that had not been exposed to filtered images.

Jang et al. (2021) proposed a noise training method, which could potentially aid artificial intelligence (AI) systems in autonomous vehicles. They found that exposure to spatially independent and spatially correlated noise increases the robustness of models to such noise, making their behavior more human like. To optimize AI-based autonomous driving systems, researchers should carefully examine what kind of visual noise is frequently present on the road and then expose the CNNs to such noise within the training protocol. These results and suggestions converge with our findings of success using the mixed-training protocol, which was motivated by how the human visual system filters incoming signals in early development.

## References

[bib1] Adeli, H., Vitu, F., & Zelinsky, G. J. (2017). A model of the superior colliculus predicts fixation locations during scene viewing and visual search. *The Journal of Neuroscience,* 37(6), 1453–1467, 10.1523/jneurosci.0825-16.2016. 28039373 PMC6705681

[bib2] Baker, N., Lu, H., Erlikhman, G., & Kellman, P. J. (2018). Deep convolutional networks do not classify based on global object shape. *PLoS Computational Biology,* 14(12), e1006613, 10.1371/journal.pcbi.1006613. 30532273 PMC6306249

[bib3] Banks, M. S., & Salapatek, P. (1978). Acuity and contrast sensitivity in 1-, 2-, and 3-month-old human infants. *Investigative Ophthalmology & Visual Science,* 17(4), 361–365. 640783

[bib4] Bar, M., Kassam, K. S., Ghuman, A. S., Boshyan, J., Schmid, A. M., Dale, A. M., … Halgren, E. (2006). Top-down facilitation of visual recognition. *Proceedings of the National Academy of Sciences, USA,* 103(2), 449–454, 10.1073/pnas.0507062103. PMC1326160 16407167

[bib5] Benedek, G., Benedek, K., Kéri, S., & Janáky, M. (2003). The scotopic low-frequency spatial contrast sensitivity develops in children between the ages of 5 and 14 years. *Neuroscience Letters,* 345(3), 161–164, 10.1016/s0304-3940(03)00520-2. 12842281

[bib5a] Bracci, S., & Op de Beeck, H. (2016). Dissociations and associations between shape and category representations in the two visual pathways. *The Journal of Neuroscience,* 36(2), 432–444, 10.1523/jneurosci.2314-15.2016. 26758835 PMC6602035

[bib6] Bracci, S., Ritchie, B. J., Kalfas, I., & Op de Beeck, H.P. (2019). The ventral visual pathway represents animal appearance over animacy, unlike human behaviour and deep neural networks. *The Journal of Neuroscience,* 39(33), 6513–6525, 10.1523/jneurosci.1714-18.2019. 31196934 PMC6697402

[bib7] Bullier, J. (2001). Integrated model of visual processing. *Brain Research Reviews,* 36(2-3), 96–107, 10.1016/s0165-0173(01)00085-6. 11690606

[bib8] Butterworth, S. (1930). On the theory of filter amplifiers. *Experimental Wireless and the Wireless Engineer,* 7, 536–541.

[bib9] Dapello, J., Marques, T., Schrimpf, M., Geiger, F., Cox, D.D., & DiCarlo, J.J. (2020). Simulating a primary visual cortex at the front of CNNs improves robustness to image perturbations. *bioRxiv*, 10.1101/2020.06.16.154542.

[bib10] Deza, A., & Konkle, T. (2020). Emergent properties of foveated perceptual systems. *arXiv*, arXiv:2006.07991.

[bib11] Dujmović, M., Malhotra, G., & Bowers, J. S. (2020). What do adversarial images tell us about human vision? *eLife,* 9, e55978, 10.7554/elife.55978. 32876562 PMC7467732

[bib12] Ellemberg, D., Lewis, T. L., Liu, C. H., & Maurer, D. (1999). Development of spatial and temporal vision during childhood. *Vision Research,* 39(14), 2325–2333, 10.1016/s0042-6989(98)00280-6. 10367054

[bib13] Firestone, C. (2020). Performance vs. competence in human–machine comparisons. *Proceedings of the National Academy of Sciences, USA,* 117(43), 26562–26571, 10.1073/pnas.1905334117. PMC7604508 33051296

[bib14] Geirhos, R., Rubisch, P., Michaelis, C., Wichmann, F. A., & Brendel, W. (2019). ImageNet-trained CNNs are biased towards texture; increasing shape bias improves accuracy and robustness. *arXiv*, arXiv:1811.12231.

[bib15] He, K., Zhang, X., Ren, S., & Sun, J. (2015). Deep residual learning for image recognition. *arXiv*, arXiv:1512.03385.

[bib16] Hermann, K.L., & Lampinen, A.K. (2020). What shapes feature representations? Exploring datasets, architectures, and training. *arXiv*, arXiv:2006.12433.

[bib17] Howard, A.G., Zhu, M., Chen, B., Kalanischenko, D., Wang, W., Weyland, T., … Adam, H. (2017). MobileNets: Efficient convolutional neural networks for mobile vision applications. *arXiv*, arXiv:1704.04861.

[bib18] Jang, H., McCormack, D., & Tong, F. (2021). Noise-robust recognition of objects by humans and deep neural networks. *bioRxiv*, 10.1101/2020.08.03.234625.

[bib19] Jozwik, K. M., Kriegeskorte, N., Storrs, K. R., & Mur, M. (2017). Deep convolutional neural networks outperform feature-based but not categorical models in explaining object similarity judgments. *Frontiers in Psychology,* 8, 1726, 10.3389/fpsyg.2017.01726. 29062291 PMC5640771

[bib20] Karras, T., Aila, T., Laine, S., & Lecthinen, J. (2018). Progressive growing of GANs for improved quality, stability, and variation. *arXiv*, arXiv:1710.10196.

[bib21] Kauffmann, L., Ramanoël, S., & Peyrin, C. (2014). The neural bases of spatial frequency processing during scene perception. *Frontiers in Integrative Neuroscience,* 8, 37, 10.3389/fnint.2014.00037. 24847226 PMC4019851

[bib22] Kiorpes, L. (2016). The Puzzle of Visual Development: Behaviour and Neural Limits. *The Journal of Neuroscience,* 36(45), 11384–11393, 10.1523/jneurosci.2937-16.2016. 27911740 PMC5125205

[bib23] Kriegeskorte, N., Mur, M., & Bandettini, P. (2008). Representational similarity analysis - connecting the branches of systems neuroscience. *Frontiers in Systems Neuroscience,* 2, 4, 10.3389/neuro.06.004.2008. 19104670 PMC2605405

[bib24] Krizhevsky, A., Sutskever, I., & Hinton, G. (2012). ImageNet classification with deep convolutional neural networks. *Communications of the ACM,* 60(6), 84–90, 10.1145/3065386.

[bib25] Kubilius J., Bracci S., & Op de Beeck, H.P. (2016). Deep neural networks as a computational model for human shape sensitivity. *PLoS Computational Biology,* 12(4), e1004896, 10.1371/journal.pcbi.1004896. 27124699 PMC4849740

[bib26] Kurihata, H., Takahashi, T., Ide, I., Mekada, Y., Murase, H., Tamatsu, Y., & Miyahara, T. (2005). Rainy weather recognition from in-vehicle camera images for driver assistance. *IEEE Proceedings. Intelligent Vehicles Symposium, 2005* (pp. 205–210). Piscataway, NJ: Institute of Electrical and Electronics Engineers, 10.1109/ivs.2005.1505103.

[bib27] Leat, S. J., Yadav, N. K., & Irving, E. L. (2009). Development of visual acuity and contrast sensitivity in children. *Journal of Optometry,* 2(1), 19–26, 10.3921/joptom.2009.19.

[bib28] LeCun, Y., Bengio, Y., & Hinton, G. (2015). Deep learning. *Nature,* 521, 436–444, 10.1038/nature14539. 26017442

[bib29] Mayer, D. L., & Dobson, V. (1982). Visual acuity development in infants and young children, as assessed by operant preferential looking. *Vision Research,* 22(9), 1141–1151, 10.1016/0042-6989(82)90079-7. 7147725

[bib30] Nguyen, A., Yosinski, J., & Clune, J. (2015). Deep neural networks are easily fooled: high confidence predictions for unrecognizable images. *arXiv*, arXiv:1412.1897.

[bib31] Norcia, A. M., & Tyler, C. W. (1985). Spatial frequency sweep VEP: Visual acuity during the first year of life. *Vision Research,* 25(10), 1399–1408, 10.1016/0042-6989(85)90217-2. 4090273

[bib32] Norcia, A. M., Tyler, C. W., & Hamer, R. D. (1990). Development of contrast sensitivity in the human infant. *Vision Research,* 30(10), 1475–1486, 10.1016/0042-6989(90)90028-j. 2247957

[bib33] Peterson, J. C., Abbott, J. T., & Griffiths, T. L. (2017). Adapting deep network features to capture psychological representations: An abridged report. *Proceedings of the Twenty-Sixth International Joint Conference on Artificial Intelligence Best Sister Conferences* (pp. 4934–4938). San Francisco, CA: International Joint Conferences on Artificial Intelligence Organization, 10.24963/ijcai.2017/697.

[bib34] Peterzell, D. H., Werner, J. S., & Kaplan, P. S. (1995). Individual differences in contrast sensitivity functions: Longitudinal study of 4-, 6- and 8-month-old human infants. *Vision Research,* 35(7), 961–979, 10.1016/0042-6989(94)00117-5. 7762153

[bib35] Petras, K., Oever, S. T., Jacobs, C., & Goffaux, V. (2019). Coarse-to-fine information integration human vision. *NeuroImage,* 186, 103–112, 10.1016/j.neuroimage.2018.10.086. 30403971

[bib36] Peirce, J. W., Gray, J. R., Simpson, S., MacAskill, M. R., Höchenberger, R., Sogo, H., … Lindeløv, J. (2019). PsychoPy2: experiments in behavior made easy. *Behavior Research Methods,* 51(1), 195–203, 10.3758/s13428-018-01193-y. 30734206 PMC6420413

[bib37] Russakovsky, O., Deng, J., Su, H., Krause, J., Satheesh, S., Ma, S., … Fei-Fei, L. (2015). ImageNet large scale visual recognition challenge. *International Journal of Computer Vision,* 115(3), 211–252, 10.1007/s11263-015-0816-y.

[bib38] Schyns, P. G., & Oliva, A. (1994). From blobs to boundary edges: Evidence for time- and spatial-scale-dependent scene recognition. *Psychological Science,* 5(4), 195–200, 10.1111/j.1467-9280.1994.tb00500.x.

[bib39] Simonyan, K., & Zisserman, A. (2015). Very deep convolutional networks for large-scale image recognition. *arXiv*, arXiv:1409.1556.

[bib40] Stiers, P., Vanderkelen, R., & Vandenbussche, E. (2003). Optotype and grating visual acuity in preschool children. *Investigative Ophthalmology & Visual Science,* 44(9), 4123–4130, 10.1167/iovs.02-0739. 12939336

[bib41] Szegedy, C., Zaremba, W., Sutskever, I., Bruna, J., Erhan, D., Goodfellow, I., & Fergus, R. (2013). Intriguing properties of neural networks. *arXiv*, arXiv:1312.6199.

[bib42] Wang, H., Wu, X., Huang, Z., & Xing, E.P. (2020). High frequency component helps explain the generalization of convolutional neural networks. *arXiv*, arXiv:1905.13545.

[bib43] Yoshihara, S., Fukiage, T., & Nishida, S. (2021). Towards acquisition of shape bias: Training convolutional neural networks with blurred images. Poster presented at Vision Sciences Society Annual Meeting [Virtual], May 21–26, 2021.

[bib44] Zang, S., Ding, M., Smith, D., Tyler, P., Rakotoarivelo, T., & Kaafar, M. A. (2019). The impact of adverse weather conditions on autonomous vehicles: How rain, snow, fog, and hail affect the performance of a self-driving car. *IEEE Vehicular Technology Magazine,* 14(2), 103–111, 10.1109/mvt.2019.2892497.

[bib45] Zeman, A. A., Ritchie, J. B., Bracci, S., & Op de Beeck, H. (2020). Orthogonal representations of object shape and category in deep convolutional neural networks and human visual cortex. *Scientific Reports,* 10(1), 2453, 10.1038/s41598-020-59175-0. 32051467 PMC7016009

[bib46] Zhou, Z., & Firestone, C. (2019). Humans can decipher adversarial images. *Nature Communications,* 10(1), 10.1038/s41467-019-08931-6. PMC6430776 30902973

